# ICO Crowdfunding: Incentives, Pricing Strategy, Token Strategy and Crowd Involvement

**DOI:** 10.1007/978-3-030-58858-8_4

**Published:** 2020-08-18

**Authors:** Gabriella Laatikainen, Alexander Semenov, Yixin Zhang, Pekka Abrahamsson

**Affiliations:** 6 IT University of Copenhagen, Copenhagen, Denmark; 7 University of British Columbia, Vancouver, BC Canada; 8 Faculty of Information Technology, University of Jyväskylä, Jyväskylä, Finland; 9 Swedish Center for Digital Innovation, University of Gothenborg, Gothenborg, Sweden

**Keywords:** ICO, Blockchain, Crowdfunding, ICOs as business models

## Abstract

Blockchain technologies provide means to develop services that are secure, transparent and efficient by nature. Unsurprisingly, the emerging business opportunities has gained a lot of interest that is realized in form of successful Initial Coin Offerings (ICOs) that are able to raise billions of USD through crowdfunding campaign. In this exploratory research we study 91 ICOs through content analysis in order to investigate the special characteristics of ICO crowdfunding as business models towards the possible investors. We found that ICOs can be described through (1) the model for providing incentives for investment, (2) the pricing strategy, (3) the token strategy and (4) the activities for crowd involvement in value co-creation.

## Introduction

The emergence of blockchain technologies disrupts industries by providing means for decentralization and making data processing more secure and more efficient [1, 2]. Furthermore, it allows both the creation and re-invention of services in different sectors by providing a decentralized environment for value creation. In a blockchain-enabled business environment, nobody has full control and lying about past events is impossible; thus, the role of regulatory actors and intermediaries disappears. Smart contracts (i.e., self-executing digital contracts) and smart properties (i.e., intelligent assets that are controllable through internet) enable the emergence of new types of businesses where organizations operate in a network with limited or no human interactions [3–5].

Initial Coin Offerings (ICOs) represent an unregulated fundraising model for startups that use blockchain technologies [1, 6]. That is, they enable projects to be funded via a crowdfunding model that can be seen as an open call for funds, evaluated and supported by a group of individuals (the crowd) [7]. To date, the most successful ICO, Filecoin was able to collect more than $257 million while ICOs raised a total of almost $11.4 billion in 2019 [8].

However, despite of their importance, ICOs are poorly understood [9, 10] and they represent a high-risk investment for investors. First, ICOs exist in an environment with no regulation, and this allows the founders to design any business model that makes their offering attractable without bearing any future consequences. Second, the underlying cryptocurrencies have a high volatility. Third, the health of the blockchain ecosystem depends on the crowd sentiments as well as it is exposed to speculations and manipulation [6, 11].

Recent literature has studied ICOs as a special type of crowdfunding (e.g. [7, 9, 10] and as revenue streams through which firms intend to collect funds for their business ideas [12]. However, in this research, we argue that ICOs can be seen as business models towards the possible investors. First, in case of ICOs the distinction between customers and investors is rather blurry. For example, in case of utility tokens the investors fund the service development for usage rights in return. Furthermore, besides a fundraising model, ICOs also incorporate many other elements that enable the founders to co-create and capture value in collaboration with possible investors. For example, ICOs often provide incentive programs through which investors have a great role in marketing and promoting the service. Thus, in this research, we look at ICOs from a business perspective and our research question is: *What are the key elements of ICOs as business models towards the possible investors?* In order to answer this question and understand the ICO phenomenon better, we collected a sample of 91 ICOs from 14 ICO enlisting sites and studied them through content analysis. The contribution of this study is two-fold. First, the study contributes to the ICO, crowdfunding and business model literature by conceptualizing ICOs as business models towards possible investors and identifying the key constituents. Second, this work has managerial implications by providing an overview of the key elements that practitioners have to make decisions on.

## Related Work

Crowdfunding represents a way to raise funds for innovative projects by linking directly capital-seeking agents and a crowd of capital-giving agents through an open call via internet [13, 14]. ICOs represent a special type of crowdfunding where founders aim to raise capital for blockchain-enabled services; thus, they can be seen as revenue models towards the crowd [12]. In ICO model, founders organize a token sale when they provide tokens or coins for the initial funders at a discounted price. These tokens may have financial value (i.e., equity or security token), functional value (i.e., utility token) and speculative value (i.e., the value resulting from the impact of token trading on the exchange of cryptocurrencies) [15, 16]. However, there are a couple of special characteristics of ICO campaigns as compared to classical crowdfunding projects. First, the tokens/coins can often be traded before the service is launched [10]. Second, in classical crowdfunding projects the founders and investors are matched via crowdfunding platforms that serve as intermediaries while ICOs are based on P2P interactions without intermediaries [14].

In the literature, different crowdfunding categories exist based on the funders’ incentives. Mollick [17] described four contexts in which individuals fund projects. First, some crowdfunding projects, such as humanitarian projects, adopt the *patronage model* where individuals donate and do not expect return. Second, in *lending model*, individuals expect some return on the capital invested. Third, in *reward-based crowdfunding*, individuals receive some kind of reward for supporting the project. The reward can be purchasing products at discounted prices, and in this way the supporters are early customers. Forth, in the *equity model*, crowdfunding supporters become investors, and they can receive equity stake for their funding.

Other categorizations of crowdfunding archetypes include the crowdfounding categories by Belleflame et al. [13]: pre-ordering and profit-sharing, and by Bradford [18]: donation, rewards, pre-ordering, lending and equity. Furthermore, Hemer identified the following crowdfunding types: donation, sponsoring, pre-ordering, membership fees, crediting, lending and profit-sharing [19].

Recent literature on ICO crowdfunding found that the investors are heterogenous and their main motives can be classified into ideology, technology, and financial motives [20]. Moreover, Fridegen et al. identified the following archetypes using cluster analysis: geographically restricted ICOs with hard funding caps and private pre-sales, geographically restricted ICOs with fiat money-oriented pricings and staking tokens, uncapped global foundation ICOs with native blockchain tokens and Global ICOs with hard funding caps [10].

## Methodology

In order to identify the special characteristics of ICO crowdfunding, we studied the business models of 91 ICOs in May and June 2018. We collected the data by running crawling scripts that gathered the name and the link of the ICOs and the category from each ICO listing site. We aimed to crawl all sites whose primary task was to enlist ICOs. After some exploratory analysis, the crawling scripts were running on the same day in the following sites: bestcoins, coingecko, coinmarketplus, icohotlist, thetokener, icorating, icomap, topicohotlist, coinschedule, icowhatchlist, icotracker, icobazaat, listico and icobench. After identifying the sample frame, we eliminated the duplicates and as a result, the data contained 4127 ICOs. Then we grouped the data by ICO categories^1^. As a final step, we identified the final data sample of 91 ICOs by choosing *randomly* at least three ICOs from each category in order to increase the industry coverage of the sample data.

We used content analysis in the data analysis phase [21]. First, we manually collected and reviewed the available information on the sample ICOs. We reviewed the ICOs websites, the whitepapers and executive summaries as well as read the information on the ICO listing sites. In the first phase of the content analysis, we identified more than 30 ICO characteristics. Then we clustered homogeneous elements and identified the key aspects that are special for blockchain-enabled businesses. In the second phase, we calculated the descriptive statistics. Some of the characteristics could not be found for each ICO. In the calculations, these missing values were not taken into account, i.e. the percentages were calculated so that 100% is the number of ICOs with available data.

During the empirical analysis, we paid special attention to the reliability and validity of the study in each step. First, the listing sites were identified and discussed by two authors. Second, the content analysis was carried out by three authors. Many ICOs were coded by two different persons. In these cases, the results were compared and discussed and the differences were negligible.

## Findings

Our study revealed that only 84% of the ICOs websites were active after a two-month period, and only 72% were active after two years. Furthermore, based on our findings, even though the quality of the whitepapers differed significantly, in general, the amount of information about the ICOs strategy, vision and operations was rather limited. The whitepapers aimed to describe the ICO’s goals and motivation, the underlying blockchain technology, the details of the ICO’s financial roadmap, the target customer segments, the key partners, the risks, etc. However, most of the whitepapers lacked some of the information, they were not transparent and not detailed enough. One of the common problems was that they did not contain clear financial roadmap or information on detailed risk assessment.

In this study we looked at ICOs as business models towards possible investors and found that they could be described through the following key characteristics: their approach for providing incentives for investment, their pricing strategy, their token strategy and the activities for crowd involvement in value co-creation. In what follows, we describe each of these elements in more details.

### Strategy for Providing Incentives for Investment: Crowdfunding Types

Based on the possible investors’ motives in our sample ICOs, we could differentiate between the following ICO crowdfunding types:

- *Equity-based ICO crowdfunding:* In this model, the investors buy shares from the business and their goal is to make profit. In this case, the majority of the tokens are allocated to the ICOs investors.
- *Rewards-Based ICO crowdfunding:* In this model, the investors get rewards, such as special usage of the service. For example, in case of ICO Wystoken the investors got discounts on the ICO’s marketplace on special products.
- *Subscription-based ICO crowdfunding:* In this model, the investors buy the possibility to use the service. For example, the ICO Oneroot provided a set of digital asset infrastructure. Their token did not provide ownership and it could not be exchanged for money; instead, buying their token gave the investors rights to use the service.
- *A combination of these:* For example, the ICO WorldTurtleCoin offered a game platform where the investors could enhance their user experience while using the ICO’s tokens in micropayments. This ICO attracted game lovers that bought tokens to use it in payments for the service (i.e., subscription-based ICO crowdfunding); however, the primary incentive for investors was to gain profit (equity-based crowdfunding).

In our data, 72% of the ICOs applied equity-based crowdfunding, 7% reward-based crowdfunding, 4% subscription-based crowdfunding and 17% some kind of combination of these.

### Pricing Strategy: Time-Based Token Valuation

The founders of ICOs organize token sales, through which they offer tokens for the possible investors. These token sales consist of Pre-Sale, Sale and Post-Sale Periods. During these periods the tokens are sold through *time-based token valuation.* This refers to a time-based second type price discrimination technique [22]. During the sale periods, the possible investors can buy the tokens for a discounted price and this price is increasing over time; thus, ICOs apply a market penetration strategy.

At operational level, ICO founders should decide on some additional properties: soft cap and hard cap, country restrictions, accepted currencies, minimum and maximum purchase. The *soft cap* refers to the minimum amount of capital that the ICO needs to gather in order to be considered as successful and to start to develop its service. On the other hand, the *hard cap* is the maximum amount of capital that the ICO aims to collect. It has to be noted that some ICOs are *uncapped* and they collect as much capital as they can. In our sample data, the greatest hard cap was about 30 millions USD.

Another operational aspect is the *country restrictions*. Some of the countries (e.g. U.S., China, Israel, Singapore) have very strict investment regulations that allows only accredited investors to participate in the ICO token sales. Thus, ICOs can choose to follow the regulations and offer the tokens only for accredited investors; or, they can open the sale for everyone and restrict the investors coming from the countries that have strict laws regarding the security of investments. However, in many cases, investors are able to find a workaround to bypass these restrictions, e.g. by using VPN. In our sample, the restricted countries included U.S. (92%), China (66%), Singapore (18%), Canada (7%) and South Korea (7%).

Another characteristic of ICOs is the *accepted currencies*. The investors can pay through different cryptocurrencies as well as, in some cases, in fiat money. In our sample data, 85% of the ICOs accepted ETH as a cryptocurrency, 45% BTC, while fiat money was accepted in 9% of the ICOs. The maximum number of accepted currencies was 45 in our data sample (ICO FeastCoin).

ICOs define the amount of *minimum and maximum purchase* that refers to the minimum and maximum amount of tokens that can be purchased. In our data sample, the maximum amount of tokens was not usually restricted. The minimum amount typically varied between 0.01 and 1 ETH (in cases that ETH was the accepted main currency - this was the case in 85% of our sample data).

### Token Strategy: Sell, Burn, Exchange, Give

The primary goal of ICOs is *selling* tokens. However, in case the hard cap is not reached, the founders typically *burn* the unused tokens or leave them *unburned*. Burning the token makes it non-existent that cannot be bought or sold anymore and thus, it reduces the total supply of available tokens. Leaving the tokens unburned returns the tokens to the founders. In some cases, ICOs redistribute the unused tokens proportionally through *Airdrop* program; thus, the tokens are given away for free. In our sample data, there was only one ICO that had an Airdrop program.

Founders may decide on the possibility to exchange the tokens into other cryptocurrencies or fiat money (i.e., *token liquidity*). Our findings reveal that most of the ICOs did not enclose detailed information regarding this in their whitepaper. In such cases there was no lock-in period but the tokens could be exchanged right after the ICO ended. In other cases, the ICOs described clearly that their tokens could not be exchanged to other currencies. These tokens could then be used only for payments for the service that the ICO developed. Finally, in some cases, the token exchange was restricted during a so-called holding period (e.g. 3 months after the ICO ends) and this temporary restriction kept away speculators.

### Strategy for Crowd Involvement in Value Co-creation: Bounties and Referral Programs

Blockchain-enabled ecosystems provide a distributed environment where the different actors co-create value. ICO founders may involve the crowd in value co-creation through *bounty* programs where they incite investors to perform small tasks and gain some reward (usually in form of tokens) in return. The bounty tasks vary greatly among ICOs; they can be related to marketing, bug reporting, development, promotion, translation, proofreading, website design, etc. We found that 60% of our sample ICOs used bounties.

Another common strategy for crowd involvement in value co-creation is the use of *referral program* as a channel through which customers are reached and targeted. ICOs typically offer the investors the possibility to gain tokens by advertising the ICO to their friends and family, or through their websites or different social media sites. The ICO benefits from this program in three ways. First, due to network effect, the value of their service increases as the number of users increase. Second, the word-of-mouth builds trust in new customers. Third, the program brings cost reduction by decreasing the marketing and advertising costs. In our sample data, 90% of the ICOs had some kind of loyalty program.

## Discussion and Conclusions

In this study, we took a sample of 4127 ICOs collected from 14 ICO enlisting sites and investigated the key aspects of 91 ICOs by analyzing the information available in the ICO enlisting websites, the ICOs’ websites and their whitepapers. We looked at ICOs as business models towards possible investors, and found that ICO founders should decide on the following key elements: (1) what incentives the ICO offers, (2) the details of the pricing strategy, (3) the token strategy, and (4) the programs to involve the crowd in value co-creation.

Related to the first element, in 72% of the cases in our sample, the possible investors had financial motives and the most used crowdfunding type was equity-based. However, some ICOs used reward-based crowdfunding by incenting investors to give funds and get some rewards in return. As a third option, some ICOs sold their tokens as a subscription for their service. That is, investors could use and pay for the services with the ICO’s own tokens.

Related to the pricing strategy, this study found that the most used pricing strategy was time-based token validation that could be seen as a market penetration strategy using second-degree price discrimination. Other operational pricing aspects consisted of soft cap and hard cap, accepted currencies, country restrictions and minimum and maximum purchase.

ICOs typically wanted to sell tokens; however, their token strategy determined also whether and under what conditions the tokens should be burned or left unburned, given for free or exchanged into fiat money (i.e., token liquidity). Finally, ICO founders typically used bounties (60% of the cases) and referral programs (90% of the cases) to incite the crowd to actively support value co-creation.

This study contributes to ICO and crowdfunding literature by conceptualizing ICOs as business models towards possible investors where the founders and investors create and capture value together. Furthermore, this research contributes to business model literature by identifying the key elements of ICOs as special type of business models. Finally, this study has managerial implications by identifying key elements that practitioners have to make decisions on.

This study is an exploratory study that has some limitations. First, the sample was collected in a limited period of time that had a limitation on the generalizability of the results because of the fast changes of the market. Second, some of the information on the websites and in the whitepapers were changed during the two-month period that the empirical study was carried out. Furthermore, the available information was not concrete and detailed enough that lead to missing values.

The research area of blockchain technology and ICOs is rather new; thus, it opens many opportunities for further research. As an example, the underlying dynamics of ICOs’ success and failures could be investigated using different research methods. Furthermore, the incentives of the ICO investors and the concept of trust in blockchain environment could be studied as well. Finally, the ICO phenomenon could be investigated from the viewpoint of IEEE’s ethical design guidelines.

## Footnotes

1 We grouped our data into the following ICO categories: Internet, Tourism, Cryptocurrency, Business services, Platform, Retail, Investment, Infrastructure, Financial services, Trading, Entertainment, Casino Gambling, Energy, Smart contract, Manufacturing, Media, Communication, Banking, Charity, Virtual Reality, Electronics, Software, Business services, Data analytics, Sports, Real estate and Health.
